# Phytase production by *Aspergillus niger* NCIM 563 for a novel application to degrade organophosphorus pesticides

**DOI:** 10.1186/s13568-017-0370-9

**Published:** 2017-03-21

**Authors:** Parin C. Shah, V. Ravi Kumar, Syed G. Dastager, Jayant M. Khire

**Affiliations:** 1 Academy of Scientific and Innovative Research (AcSIR), Council of Scientific and Industrial Research-National Chemical Laboratory, CSIR-NCL, Pune, 411008 India; 2 National Collection of Industrial Micro-organisms (NCIM) Resource Center, Biochemical Sciences Division, CSIR-NCL, Pune, 411008 India; 3 Chemical Engineering and Process Development Division (CEPD), CSIR-NCL, Pune, 411008 India

**Keywords:** *Aspergillus niger*, Green chickpea flour, Fermenter scale, Organophosphorus pesticide, Phytase, Submerged fermentation

## Abstract

The production of phytase using *Aspergillus niger* NCIM 563 under submerged fermentation conditions was studied using protein rich chickpea flour as substrate. Employing a hybrid statistical media optimization strategy of Plackett–Burman and Box-Behnken experimental designs in shake-flasks gave an increased phytase activity from an initial 66 IU/mL in 216 h to 160 IU/mL in a reduced time of 132 h. Productivity, thus increased by 3.97 times from 7.3 to 29 IU/mL/day. Using the optimized media, the production was successfully scaled-up further and improved up to 164 IU/mL in 96 h by studies carried out employing 2 and 10-L fermenters. The enzyme supernatant was recovered using centrifugal separation of biomass and the stability of the produced phytase was tested for animal feed applications under gastric conditions. In vitro degradation studies of water soluble monocrotophos, methyl parathion and water insoluble chlorpyrifos, pesticides used extensively in agriculture was carried out. It was observed by HPLC analysis that phytase could degrade 72% of chlorpyrifos at pH 7.0, 35 °C. Comparable results were obtained with monocrotophos and methyl parathion. With chlorpyrifos at higher temperature 50 °C as much as 91% degradation could be obtained. The degradation of chlorpyrifos was further validated by spraying phytase on harvested green chilli (*Capsicum annuum* L) under normal conditions of pH 7.0, 35 °C and the degradation products obtained analyzed by LCMS. Thus, the present study brings out a potentially novel application of phytase for biodegradation of organophosphorus pesticides.

**Electronic supplementary material:**

The online version of this article (doi:10.1186/s13568-017-0370-9) contains supplementary material, which is available to authorized users.

## Introduction

Over the past century, an increase in demand of food grains and vegetables has led to an extensive use of manmade pesticides in agriculture. In fact, crop protection in India is known to annually use nearly 40,000 metric tons of pesticides. Organophosphorus pesticides (OpP), are widely used in agriculture for controlling variety of sucking, chewing and boring insects, spider mites, aphides and pests. In particular, OpP cannot be easily removed by washing and rinsing with tap water (Vendan 2016) and this leads to bioaccumulation in the food chain.

Organophosphorus pesticides are esters of phosphoric acid, which include aliphatic, phenyl and heterocyclic derivatives (Baishya and Sarma 2015), which are known to be potent irreversible acetylcholinesterase (AChE) inhibitors by phosphorylation of the serine residue at the enzyme active site. This leads to adverse effects on the nervous system of exposed animals including humans (Mileson et al. 1998), and there exists a dire need to degrade OpP post-harvest, so as to prevent their entry into the food chain.

The currently used physicochemical processes for OpP remediation include incineration or disposal in landfills that are expensive, non-ecofriendly and the process is often incomplete leading to formation of toxic intermediates (Debarati et al. 2005). Alternatively, the use of whole-cell microorganisms is advantageous because it offers a safe, economic and eco-friendly green option (Rayu et al. 2012; Sutherland et al. 2004). The factors that impact bioremediation are mainly the availability of organic sources for microbial growth, optimal pH, bioavailability of inhibitory substrates and the satisfaction of regulatory norms for release of microbes into the environment (Boopathy 2000), which can be addressed by using cell-free microbial enzymes that can act on diverse pollutants (Scott et al. 2011).

Most widely studied cell-free enzymes for OpP degradation are from bacteria, viz., organophosphorus hydrolase (EC 8.1.3.1) (Gao et al. 2012), phosphotriesterase (EC 3.1.8.1) (Chino-Flores et al. 2012) and organophosphorus acid hydrolase (EC 3.1.8.1) (Theriot and Grunden 2011). There are few reports where, fungi have been studied for OpP degradation by cleaving the phosphate group (John and Shaike 2015; Wyss et al. 1999). Phytase (PYT) or myo-inositol hexakisphosphate phosphohydrolase (EC 3.1.3.8) from *Aspergillus niger* NCIM 563, is a good example of a hydrolytic enzyme that can release inorganic phosphorus by the degradation of phytic acid (Bhavsar et al. 2008). Current progress on PYT research is focused on phytic acid degradation for major animal feed supplementation, plant growth promotion and human nutrition (Dersjant-Li et al. 2015; Kumar et al. 2010). The GRAS cleared PYT enzyme has not been studied for its potential for biodegradation of toxic pesticides by phosphorous release and is therefore studied here. A positive result would bring out a novel and useful enzymatic application of PYT for pesticide detoxification.

Agricultural residues including rice bran, wheat bran, groundnut oil cake, corn starch, etc., are widely used as substrates for PYT production (Bhavsar et al. 2008; Alves et al. 2016; Buddhiwant et al. 2015). However, the cost of production increases with use of agricultural residue as it needs pre-treatment (Bhavsar et al. 2008; Rani et al. 2014). The need for cost effective sustainable production requires alternative substrate for PYT production. In this context, the use of protein-rich legume flour as substrate has advantages for sustainable low cost PYT production because it can avoid the pre-treatment step. India is the largest producers of chickpea (*Cicer arietinum*) (7.17 metric tons in 2014–2015) but around 20% of the cultivated chickpea seeds are rejected due to non-uniform growth, color and damage during harvesting and post-harvesting process (Torres-Fuentes et al. 2011). These rejected green seeds are however, rich in protein, carbohydrate, lipids and major dietary minerals such as calcium, phosphorus, magnesium, iron and potassium (Christodoulou et al. 2006) and has been used in poultry diet (Garsen et al. 2007). For the above reasons, in the present study, in view of its availability as agricultural waste, we used green chickpea flour (GrCf) as the substrate of choice for producing and maximizing PYT production. The increased production of PYT would facilitate studying for animal feed applications and as discussed earlier in conducting studies related to dephosphorylation potential of OpP by this extracellular enzyme.

In the present study for increasing the production of extracellular PYT from GrCf using *A. niger*, growth media optimization was envisaged using shake flasks under submerged fermentation condition (SmF) by employing an effective hybrid strategy that involves carrying out statistical sets of experiments (Shah et al. 2009). In this approach, we initially aim at identifying the significant factors influencing PYT production by implementing a Plackett–Burman Design (PBD) of experiments (Plackett and Burman 1956). Subsequently, a second set of experiments can then be conducted to further optimize the levels of the significant factors that maximize PYT production by application of a more rigorous experimental design such as the Box Behnken design (BBD) (Box et al. 1978; Khuri and Cornell 1987). Obtaining positive results would then suggest scale-up studies with 2 and 10-L fermenters so as to confirm maintenance or obtain improvements in the production levels for process feasibility. This would also require assessing the suitability and stability of the PYT produced by GrCf so that animal feed applications can become possible. As discussed above, from a novel application point of view, it would be interesting to study and test the enzyme effectiveness for in vitro pesticide degradation using for example a commercially available OpP such as water insoluble chlorpyrifos (CPyF) (Dursban 2E©) and water soluble monocrotophos (MCP) and methyl parathion (MP). Again, a positive result would suggest carrying out studies with a test system such as post-harvest fresh green chillies (*Capsicum annum* L) treated with CPyF. The results obtained by carrying out suitable studies for the above objectives and plan are discussed in this work.

## Materials and methods

### Chemicals

Phytic acid sodium salt, 3, 5, 6-trichloro-2-pyridinol (TCP) and diethyl thiophosphate (DETP) was purchased from Sigma Chemical Company (St. Louis, MO, USA). Acetonitrile (ACN) of HPLC grade was purchased from Merck. All other chemicals used were of analytical grade. CPyF (Dursban 2E©, 20%), MCP (36%) and MP (50%), harvested green chilli, soybean meal and seeds of green chickpea were purchased from a local market. The seeds were minced in a grinder to obtain green chickpea flour (GrCf).

### Microorganism and production in basal media

*Aspergillus niger* NCIM 563, used in the present study was obtained from National Collection of Industrial Microorganisms (NCIM), CSIR-National Chemical Laboratory (CSIR-NCL), Pune, India, which was maintained on potato dextrose agar (PDA) slants and stored at 4 °C. A time course of the PYT production was studied using the basal media (pH 5.5) in triplicates. 100 mL media comprising of (g%): 1.0 GrCf; 5.0 glucose; 0.86 NaNO_3_; 0.05 KCl; 0.05 MgSO_4_·7H_2_O; 0.01 FeSO_4_·7H_2_O was dispensed in 250 mL Erlenmeyer flask and sterilized by autoclaving at 121 °C for 20 min. Spores from 7 days old PDA slant were gently scraped using sterile wire loop with 25 mL sterile saline solution containing 0.01% Tween 80. The spore suspension was collected in sterile tube and homogenized by vortexing for 1 min. 1 × 10^7^ spores (using Neubaur chamber) was used as spore inoculum (Sp-I) for inoculating the basal media and incubated at 28 °C at 170 rpm under aerated culture conditions. Vegetative inoculum (Ve-I) was prepared by inoculating 5 mL basal media with 1 × 10^7^ spores under same aerated culture conditions for 10 h which was used for inoculating 95 mL basal media. The samples were withdrawn every 24 h and centrifuged. The supernatant was checked for total residual glucose and PYT activity.

### PYT assay

The PYT analysis solution consisting of 3 mM sodium phytate with 100 mM glycine–HCl buffer (pH 2.5) and 100 μL of liquid enzyme extract solution was incubated for 30 min at 50 °C. The liberated inorganic phosphate was measured by the ammonium molybdate method (Heinohen and Lathi 1981). A freshly prepared solution of acetone, 5 N H_2_SO_4_, 10 mM ammonium molybdate (2:1:1, v/v) and 400 μL citric acid (1 M) was added to the enzyme-substrate solution and absorbance was measured at 370 nm against blank consisting of buffer and substrate. One unit of PYT activity (IU) was expressed as the amount of enzyme that liberates 1 μmol phosphorus/min/mL under standard assay conditions while enzyme production was expressed as PYT activity IU/mL.

### Stability studies

The influence of pH and temperature on PYT activity was determined by assaying in the pH range of 1.5 and 9.0 using 100 mM buffers: glycine–HCl (pH 2.0–3.0), sodium acetate (pH 4.0–6.0), Tris–HCl (pH 7.0–8.0), and glycine–NaOH (pH 9.0) at 50 °C. The pH stability using the same buffer solutions was also determined by pre-incubating enzyme samples at 35 °C for the period of 12 h considering PYT activity at zero time as 100%. The studies on optimum temperature were carried out in the temperature range of 30–70 °C, while the temperature stability was determined by incubating the enzyme samples over the above temperature range for a period of 1 h on comparing with the control without incubation. The stability of PYT was also checked at gastric conditions of poultry. One gram soybean meal was dissolved in 9 mL of simulated gastric fluid (250 mM glycine–HCl containing 2.0 mg/mL NaCl and 3.2 mg/mL of pepsin) and the pH was adjusted over a range from 1.5 to 6.5 using HCl and NaOH as required. The solutions were incubated at 37 °C for 30 min, as the poultry gut temperature varies from 37–39 °C (Lei and Stahl 2001). 40 IU of PYT was added to the solution and incubated at 37 °C for 60 min. The released phosphorus was determined as described in PYT assay.

### Biodegradation of OpP using PYT

The potential of extra-cellular PYT, produced in basal media, was studied for biodegradation of CPyF, a water insoluble organophosphate. 1 mL stock solution of CPyF (10,000 ppm) was incubated with 100 µL mycelial free PYT (100 IU; specific activity 53 IU/mg) for 2 h at 35 °C, pH 7 as well as its optimum conditions (50 °C, pH 2.5). The selection of a high concentration of CPyF was employed for sensing and quantifying the degradation metabolites. The treated sample was diluted 10 times with mobile phase and the amount of residual CPyF was monitored using HPLC as mentioned in analytical methods. For other OpPs (MCP and MP), studies were carried out in similar way.

### Analytical methods

Concentration of the protein (mg) was determined using Lowry method with bovine serum albumin as standard (Lowry et al. 1951) and specific activity (IU/mg) using enzyme units and protein concentration were calculated. Di-nitro salicylic acid (DNSA) method (Miller 1959) was used to calculate total residual reducing sugar concentration.

The analysis of OpPs were carried out using HPLC (Dionex-ASI 100, auto sampler series) with reverse-phase column (C18 −4.6 × 250 mm, Waters) maintained at 40 °C. The mobile phase used was mixture of ACN: water (70:30 v/v), with flow rate of 0.5 mL/min. The detection was done at 230 nm and injection volume was 50 µL. Standard solutions of OpP was transferred in vials to reach final concentrations in range of 100–1000 ppm using mobile phase. The analysis of CPyF and its degraded products on green chilly were carried out using LC–MS (Waters-Xevo TQD-USA) with reverse phase C18 column (Acquity—UTLC BEH—2.1 × 100 mm) maintained at 45 °C. The mobile phase used was mixture of ACN: water (70:30 v/v), with flow rate of 0.3 mL/min. Injection volume of sample was 5 µL. Standard solutions of each was transferred in vials to reach concentration in range of 0.2–1 ppm using mobile phase.

### Application of PYT on harvested green chilli (*Capsicum annuum* L)

The potential of PYT to degrade CPyF on harvested green chilly was studied at 35 °C, pH 7.0. Green chillies (250 g), obtained from local market was sprayed with CPyF (20 ppm) and dried. One part of the chillies (test) was treated with crude mycelial—free PYT (80 IU) at 35 °C and pH 7.0 for 2 h keeping the second part untreated (control). Both the parts were separately cut into small pieces and homogenized with a household mill (equipped with stainless steel knives). 10 mL of ACN was added to 10 ± 0.1 g sample and vortexed for 1 min to which 10 g sodium sulfate was added and homogenized at 15,000 rpm for 1 min. The samples were centrifuged at 5000 rpm for 5 min and 5 mL of the supernatant were transferred to a 15 mL PTFE tube. 10 mg graphitized carbon black and 25 mg primary secondary amine were added to remove colored impurities. The extract was shaken using a vortex mixer for 30 s and centrifuged at 10,000 rpm for 5 min. 2 mL of the supernatant was used to analyze the presence of CPyF and its degraded metabolites using LC–MS as mentioned in analytical methods.

### Media optimization in shake flasks

Carrying out a PBD of experiments offers a rapid multifactor way to screen and identify the most significant factors (Plackett and Burman 1956). The potential effect of 10 variables (Additional file 1: Table S1) on PYT production, were evaluated in 12 PBD runs at two levels, low level (−) and high level (+). The choice of the above variables was made based on reports available for enhanced PYT production by solid state fermentation (Bhavsar et al. 2013). The complete PBD matrix for screening was designed using a standard Plackett–Burman orthogonal array constructed using Design Expert Software (DES) Version 7.1.2, Stat-Ease, Minneapolis, MN, USA. The response values of PYT produced in IU/mL were analyzed to obtain a best-fit linear mathematical model that could be further analyzed by ANOVA for acceptability. Subsequently, a BBD of experiments was generated by DES and studies at three different levels −1, 0, +1 (Additional file 1: Table S2) were carried out to further optimize enzyme production levels with respect to the major factors identified by PBD. The less significant factors were maintained at the average of the high and low levels used in the PBD study. A best-fit model for enzyme production was further studied by ANOVA to test for its statistical significance.

### Fermenter scale production

Scale-up studies in a batch fermenter (New Brunswick BioFlo 110) with 2-L media was carried out on the basis of optimized media formulation. Successive fermentation batches at different rpm (400, 500 and 600 rpm) were performed with constant aeration of 0.5 vvm and temperature of 28 °C. The fermenter containing 1.8-L medium that was earlier sterilized in situ was inoculated with 36 h old Ve-I (200 mL). The dissolved oxygen (DO) was measured using Mettler Toledo oxygen probe. Samples from the fermenter were withdrawn at regular intervals and analyzed for PYT activity and residual glucose on biomass separation. The process was further scaled up using a 10-L capacity fermenter with 1-L, 36 h old Ve-I.

## Results

### PYT production in basal media

**Fig. 1:**
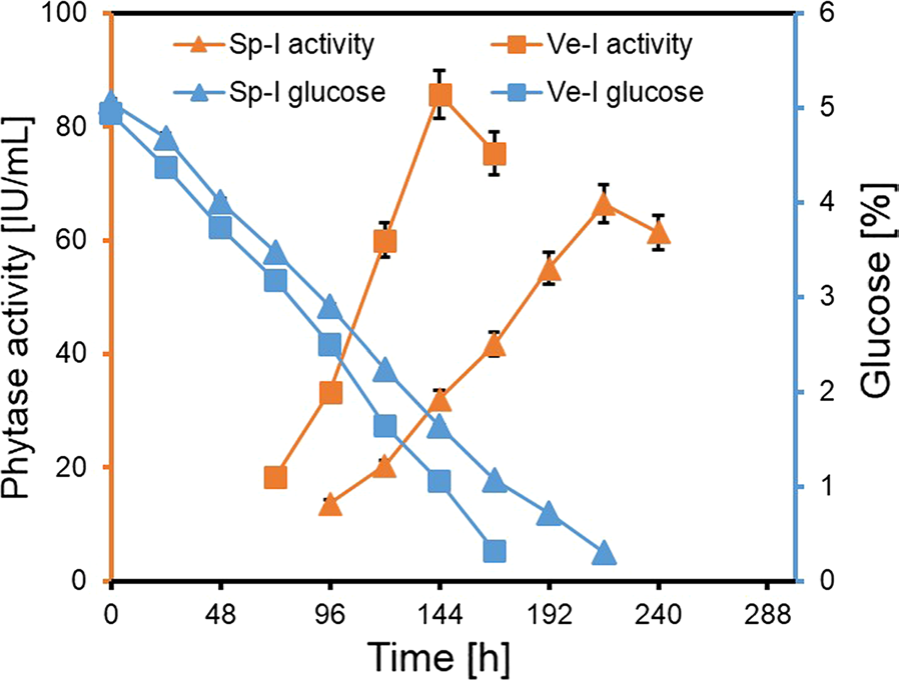
Comparison of phytase production using spore inocula (Sp-I) and vegetative inocula (Ve-I). Experiments were carried out in triplicate (mean ± SD)

The PYT production was tested using two types of inoculum; spore (Sp-I) and vegetative (Ve-I). The results showed, slow and gradual increase in PYT production using Sp-I, with the maximum activity of 66 ± 3.3 IU/mL on 9th day, (i.e., 216 h) (Fig. 1). The glucose concentration also showed a gradual depletion, with complete utilization by 10th day (i.e., 240 h). In fact, using Ve-I, higher PYT activity of 86 ± 4.3 IU/mL was observed in a lower production time of 6 days (i.e., 144 h) with glucose depletion in 7 days (i.e., 168 h). Thus, the productivity [units of enzyme produced per day (IU/mL/day)] increased from 7.3 IU/mL/day obtained using Sp-I to 14.3 IU/mL/day using Ve-I. Thus, the type of inoculum has marked effect on the production of PYT and all further studies were therefore carried out using Ve-I.

### Stability studies of PYT

The conditions for optimum activity and stability of the PYT was assessed by carrying out studies with respect to temperature and pH. Optimum temperature studies ranged from 30 to 70 °C. It was observed that, the optimum temperature for maximum PYT activity is 50 °C (Additional file 1: Figure S1). On considering the activity at 50 °C to be 100%, we observe that at 35 °C, the activity reduces to 30% of maximum activity and at 60 °C to 60% of maximum activity. Temperature stability studies were then carried out, which showed 100% stability in 1 h from 30 to 50 °C (Additional file 1: Figure S1) while 20% activity was reduced at 60 °C, 50% at 65 °C and 100% at 70 °C. Studies with varying pH interestingly showed that the enzyme showed high activity at a low pH value of 2.5 (Additional file 1: Figure S2). Considering the activity at pH 2.5 to be 100%, only 50 and 10% activity was observed at pH 4.5 and 7.0, respectively. It may be noted that the PYT showed overall broad pH stability from pH 2.5–9 (Additional file 1: Figure S2). Experiments showed that the enzyme activity was retained for 12 h in the pH range studied. The pH and temperature stability profile was also determined under poultry gut conditions as described in methods. High efficacy of phosphate release was shown by the PYT in simulated gastric fluid in pH ranging from 2.0 to 4.5 (Additional file 1: Figure S3).

### Biodegradation of OpP using PYT

**Fig. 2:**
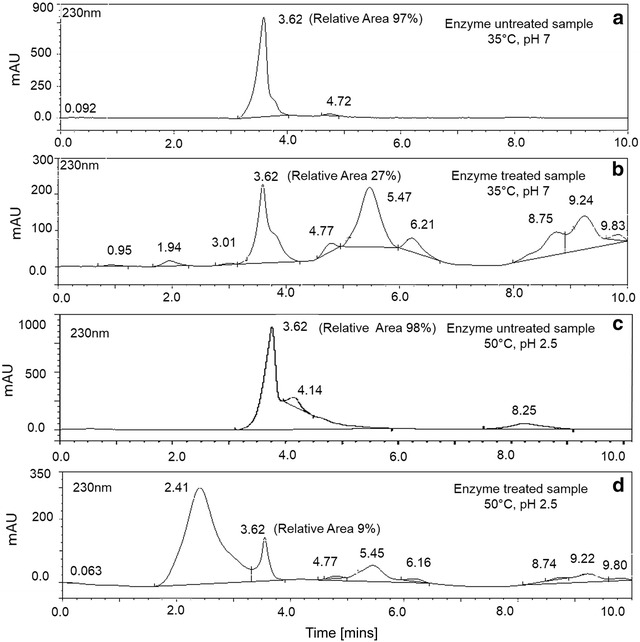
Reduction of CPyF using phytase at different conditions **a**, **c**. CPyF before phytase action, **b**, **d**. CPyF after phytase action

The ability of crude PYT, (100 IU) produced using basal media, to degrade CPyF was studied both under normal conditions (35 °C, pH 7.0) as well as under optimum enzyme conditions (50 °C, pH 2.5). HPLC analyses, showed a single major peak for the sample containing only CPyF (control sample) at 35 °C, pH 7.0 (Fig. 2a) as well as at 50 °C, pH 2.5 (Fig. 2c) and having a retention time of 3.62 min with a relative area of 97%. On the other hand, for a PYT treated sample at 35 °C, pH 7.0, multiple peaks were observed. Notably, it was observed that there was decrease in relative area by 72% at the retention time of CPyF (Fig. 2b). A similar study with PYT at 50 °C, pH 2.5, the relative peak area seen at retention time of CPyF showed an even higher decrease in relative area by 91% (Fig. 2d). The positive result for CPyF suggested degradation studies with other OpPs, namely, MCP and MP by PYT would be useful. HPLC analysis with MCP having a retention time of 5.5 min for the control sample (Additional file 1: Figure S4a) at 35 °C, pH 7.0 showed that a higher unit of PYT (250 IU) to obtain 53% degradation in 4 h (Additional file 1: Figure S4b) when compared to CPyF (PYT 100 IU, 72% degradation) (Fig. 2) in 2 h. Better degradation results with MP (HPLC retention time of 9.5 min for the control sample, Additional file 1: Figure S4c) in comparison with MCP were obtained by a higher decrease in peak area (77%) on treatment with PYT (250 IU) (Additional file 1: Figure S4d) in 4 h. Thus, all the above results with CPyF, MCP and MP corroborate the finding that PYT has the ability to effectively act on OpP and degrade them.

### Application of PYT on harvested green chilli (*Capsicum annuum* L)

Phytase shows dephosphorylation action by breaking the phospho-ester bond to release phosphate from substrate (Joshi 2014). As per food safety and standards authority of India (FSSAI), limit of CPyF on vegetables is 0.2 ppm (FSSAI notification 2011). Detoxification of CPyF is achieved by cleaving the phospho-ester bond, generating TCP as the major product along with DETP (Chen et al. 2012; Hanley et al. 2000; Bicker et al. 2005), which are both water soluble. Human studies show that, both the metabolites are considered as urinary markers of CPyF exposure and are easily excreted through urine within 12 h. Studies on rat show that, TCP and DETP are the predominant urinary metabolites of CPyF catabolism (Bicker et al. 2005). In the present study, the applicability of PYT applied on post-harvest chillies to degrade water insoluble CPyF was therefore studied with respect to the formation of TCP and DETP water soluble degradation products.

**Fig. 3:**
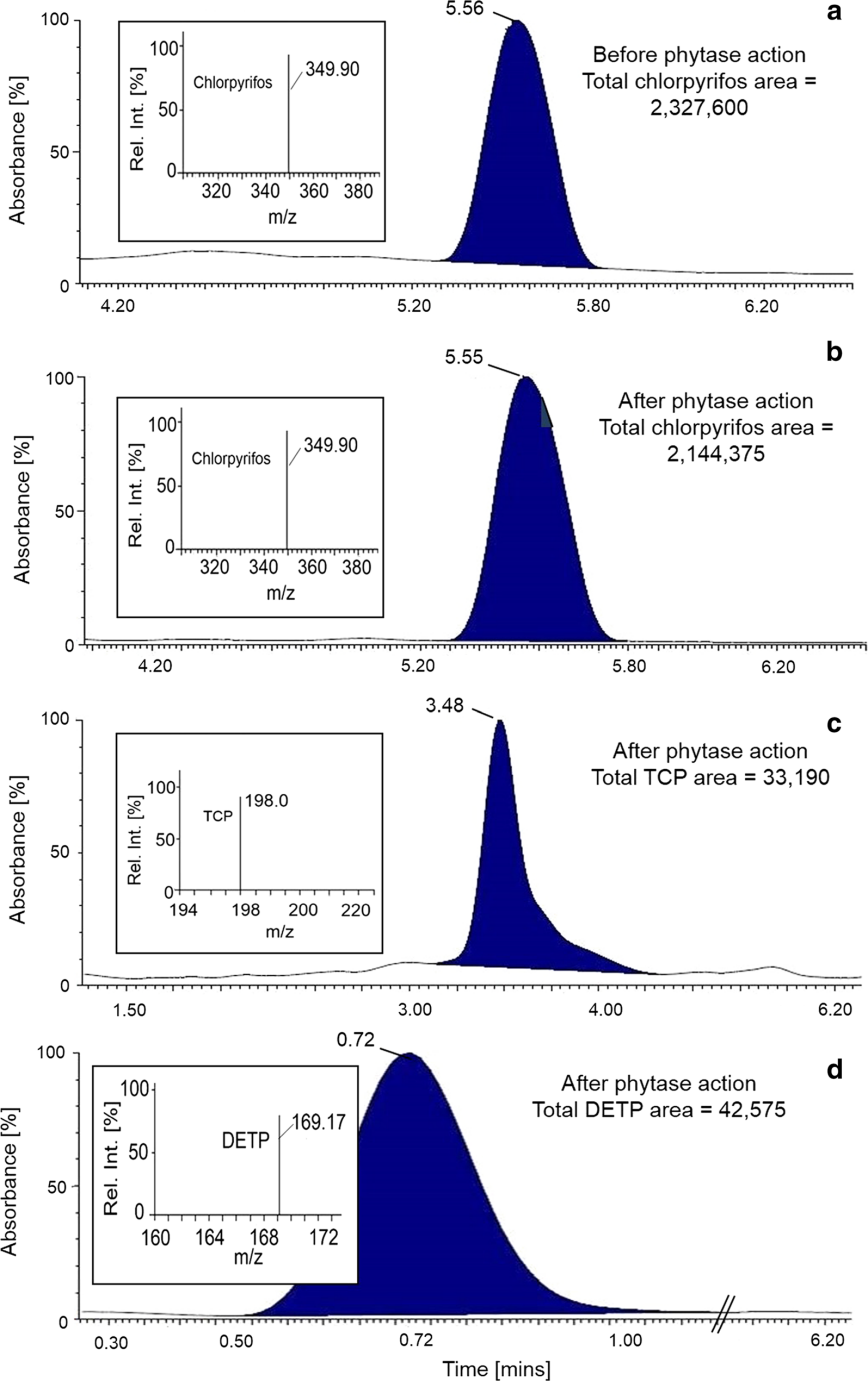
Analysis of CPyF and its degraded metabolites using LC–MS. **a** CPyF on green chilli before phytase action. **b** CPyF on green chilli after phytase action. **c** Release of TCP after phytase action. **d** Release of DETP after phytase action

LC–MS analysis of standard CPyF showed that it eluted with a retention time (RT) of 5.56 min and *m/z* of 349.90 while DETP and TCP eluted at 0.72 and 3.48 min with *m/z* values of 169.17 and 198, respectively. In PYT untreated sample (control), a single peak was detected after LC–MS analysis with a RT of 5.56 min and *m/z* of 349.90 indicating it to be CPyF (Fig. 3a). In PYT treated sample, 3 peaks (Fig. 3b–d) were observed at RT of 0.72, 3.48 and 5.56 min. MS analysis of these peaks showed *m/z* values of 169.17, 198 and 349.90, respectively. On comparing with the standards, the 3 peaks were ascertained to be DETP, TCP and CPyF, respectively. The percent degradation of CPyF using PYT from *A. niger* NCIM 563 can vary depending on pH and temperature prevailing at the field. Peak area analysis shows 8% degradation of CPyF at 35 °C and pH 7.0 in 2 h using PYT (80 IU). To increase the degradation, higher units of phytase or reaction time may thus be required. On using higher units of PYT (250 IU) to degrade CPyF on green chilli our results in fact showed that 90% degradation was possible in 12 h (Additional file 1: Figure S5a, b). It is reported that TCP is not fetotoxic and teratoxic in either rat or rabbits at dosage levels of 100 ppm. TCP is shown to have moderate toxicity to salmonoids at LD_50_ value of 1.8 ppm (Marino et al. 1999). Studies of TCP showed that a minimum concentration of 0.6 ppm when exposed for 24 h is toxic during the multiple developmental stages of zebra fish (Suvarchala and Philip 2016). Our observation is that PYT can degrade CPyF present on raw agricultural products. Thus, development of a potential new way that prevents toxic OpP from entering the food chain by forming easily removable metabolites could become possible.

### Media optimization for enhanced PYT production in shake flask

For the 12 PBD runs the experimentally obtained maximum response values of PYT activity (IU/mL) obtained on the 6th day (i.e., 144 h) are reported in Additional file 1: Table S3. Run number 10 showed a maximum PYT production value of 132 ± 6.6 IU/mL. Regression analysis of the response values obtained for the PBD runs yielded a best fit linear model, viz.,

1 $$\begin{aligned} {\text{Phytase activity }} = { 111}.0 9 - 30. 6 7 { } \times \left[ {{\text{NaNO}}_{ 3} } \right] + 4 5. 3 3\times \left[ {\text{GrCf}} \right]{-} 1 6 4 4. 4 4 { } \times \left[ {{\text{MnSO}}_{ 4} \cdot{\text{H}}_{ 2} {\text{O}}} \right] \, \hfill \\ \, {-} 1 90.0 \times \left[ {{\text{CaCl}}_{ 2} \cdot 2 {\text{H}}_{ 2} {\text{O}}} \right] \hfill \\ \end{aligned}$$

The suitability of the model was further corroborated by ANOVA tests. Thus, the obtained model F-value of 11.35 implies the model is significant and that there is only a 0.35% chance that the model F-value could occur due to noise. The values of Prob > F was less than 0.05, for the four variables showing their significance. The coefficient of determination R^2^ = 0.87 provided a satisfactory measure for the variability in the observed response that could be explained by the model. The Pred R^2^ of 0.61 is in reasonable agreement with Adj-R^2^ of 0.79. The adeq precision, a measure of the signal-to-noise ratio, is found to have a high value of 9.72 and this indicated the signal strength to be strong. All the above tests confirm that the model Eq. (1) can be used to navigate the design space. In fact, using the above model, it was found that, the above four factors accounted for 86.68% of the total contribution to the estimates of the response values. The remaining variables then accounted for only 13.32% and thus PBD identified them to be less significant. The ANOVA results were complemented by the fact that four out of the 10 factors studied in the PBD, namely, NaNO_3_, GrCf, MnSO_4_·H_2_O and CaCl_2_·2H_2_O were significant on comparing their t-values using a Pareto chart (Additional file 1: Figure S6).

The maximum contributory factors identified by PBD for PYT production were further optimized by a BBD of experiments (29 runs) generated by DES employing three chosen levels for each variable. Additional file 1: Table S4 gives the BBD for the four significant variables along with the experimentally obtained PYT activity. We observe that the optimization studies showed that run number 25 remarkably improved the PYT activity (160 ± 8.0 IU/mL). The wide variation in activity reported in all the runs bring out the process sensitivity to the experimentally chosen conditions and shows the usefulness of having carried out this systematic optimization study. The response data of BBD runs was regressed successfully using actual factors and interestingly showed linear dependency without interacting terms, namely,

2 $${\text{Phytase activity}} = 2 8 6. 7 4 - 4 9. 1 7\times \left[ {{\text{NaNO}}_{ 3} } \right]{-} 1 20. 8 3\times \left[ {\text{GrCf}} \right]$$

The ANOVA analysis of the above model satisfied the statistical tests with an obtained model F-value of 27.95 implying that, the model is significant with only a 0.01% chance that the model F-value could arise due to noise. The value of correlation coefficient (Pred R^2^ = 0.62) for PYT production suggested a good agreement between the observed and model predicted response values. The coefficient of determination (R^2^ = 0.68), suggests that 68% of the variability in the data was explained by Eq. 2. The obtained signal-to-noise ratio value of 15.04 brought out the presence of an adequate signal.

**Fig. 4:**
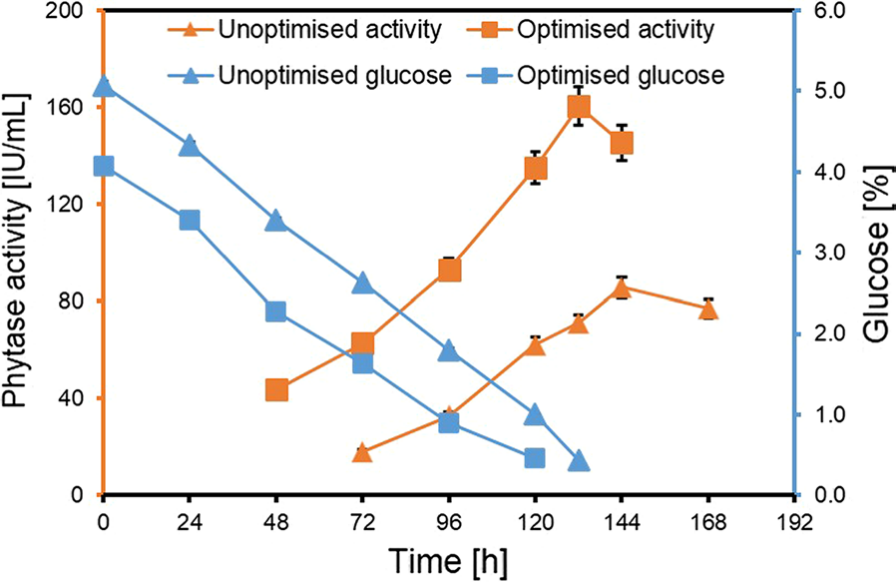
Validation of phytase production by using unoptimized and optimized media. Experiments were carried out in triplicate (mean ± SD)

The final formulation of ten variables in the range studied in PBD and BBD showed that the highest activity of 160 ± 8.0 IU/mL was obtained in 132 h with 100 mL media comprising of (g%): 4.0 glucose; 0.4 NaNO_3_; 0.075 MgSO_4_·7H_2_O; 0.075 KCl; 0.015 FeSO_4_·7H_2_O; 0.015 Tween 80; 1.0 GrCf; 0.35 dextrin; 0.02 MnSO_4_·H_2_O and 0.1 CaCl_2_·2H_2_O. To validate the formulation, a time course experiment for PYT production was carried out using this optimized condition. The results obtained after showed that a 1.86 fold enhancement in PYT activity from 86 ± 4.3 IU/mL to 160 ± 8.0 IU/mL with glucose completely utilized in 5.5 days (i.e., 132 h) was achieved by adopting the outlined hybrid media optimization approach (Fig. 4). The effect of varying the phosphate concentration by addition of sodium phytate and KH_2_PO_4_ using the optimized media formulation with 1% GrCf was thus studied. Results showed that the PYT activity decreased to 79 IU/mL on addition of sodium phytate (0.004 g%) and to 151 IU/mL on addition of KH_2_PO_4_ (0.002 g%). This may be compared to PYT activity of 160 IU/mL obtained using the optimized media with 1% GrCf and suggests that it optimally provides the requirements of phosphate.

### Scale-up studies of PYT production in batch fermenters

**Fig. 5:**
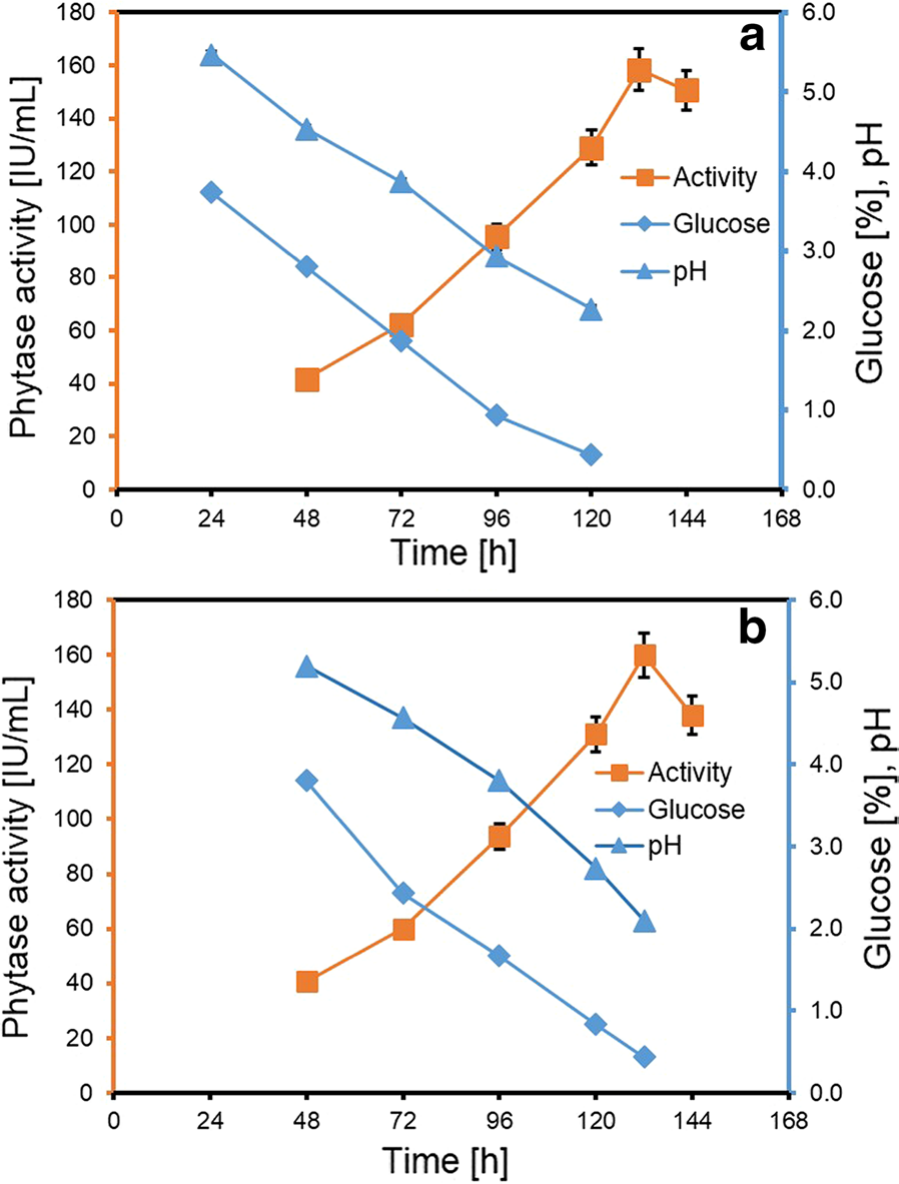
Fermenter scale production **a** 2-L (at 600 rpm) and **b** 10-L (at 500 rpm) depicting phytase activity, pH and glucose concentration. Experiments were carried out in triplicate (mean ± SD)

The optimized media formulation was studied in scaled-up larger volume batches using fermenters to ensure maintenance of the PYT production throughput. The results of fermentation studies carried out with 2 and 10-L media scaled-up volume batches are discussed below. It is expected that when operating with larger volumes, the dissolved oxygen (DO) is an important factor to consider and this would depend on both the aeration rate and the agitation speed. For the present studies, we chose to keep the aeration rate constant at 0.5 vvm while varying the initial agitation speeds (400, 500, 600 rpm). The behavior in time of DO and pH were simultaneously monitored. For the 400 rpm run, a decrease in DO was observed till 36 h. 50% DO was maintained by gradual increase in rpm and it was thus maintained at the higher rpm. The pH of the media gradually decreased from 5.0 to 2.3. For the 2-L batch study with an initial rpm of 400, the maximum activity of 97 ± 4.8 IU/mL was obtained in 132 h. PYT production of 122 ± 6.1 IU/mL and 158 ± 7.9 IU/mL was achieved at initial rpm of 500 and 600 respectively, in 132 h. Thus, PYT production was successfully scaled up to 2-L production from the shake flask experiment (158 ± 7.9 IU/mL at 600 rpm) (Fig. 5a).

Successful production at 2-L scale, paved way to studying the feasibility of enzyme production in a 10-L volume fermenter to bring out the process biotech potential. Similar to studies in the 2-L scale and varying the initial agitation speed (400, 500 and 600 rpm), the DO and pH were monitored in 10-L scale. DO and pH pattern were again observed, but with higher decreasing rates than in the 2-L scale. Remarkably, the maximum PYT activity of 164 ± 8.2 IU/mL was maintained but it is important to note that the maximum activity was obtained in a much shorter time of 96 h at 500 rpm (Fig. 5b).

## Discussion

Phytase is widely used to act on phytic acid for the aim of increasing the bioavailability of phosphorus, proteins and essential minerals in animal diets. It is commercially produced by employing a submerged fermentation process using spore inoculum (Coban and Demirci 2014; Krishna and Nokes 2001). We considered it desirable to carry out studies that use vegetative inoculum for PYT production over spore inoculum that is commonly employed for PYT production. Results obtained suggest that PYT production may in fact be enhanced using vegetative inoculum. This result is of considerable significance. Similar results of enhanced PYT production have been observed using vegetative inoculum in solid state fermentation (Krishna and Nokes 2001). Our studies also show that the use of GrCf as substrate for PYT production with *A. niger* NCIM 563 gives activity and productivity higher than earlier reports under submerged fermentation conditions using different strains and substrates (Table 1). Our observation is that the PYT produced using GrCf is active and highly stable over wide range of temperature and pH that simulates gastric conditions and brings out its superiority for use in animal feed applications. A novel application of PYT in degrading CPyF, MCP and MP is also discussed here to bring out its potential as an agent for pesticide degradation. It needs to be noted that FDA has approved *A. niger* as a generally-recognized-as-safe (GRAS) organism (Schuster et al. 2002). Together with the laboratory study and analysis report obtained from Central Avian Research Institute, Bareilly (UP), India confirms that *A. niger* NCIM 563 is a non-mycotoxin producer and the PYT obtained can thus be advantageously exploited.

**Table 1:** Comparing phytase productivity of *A. niger* NCIM 563 with other *Aspergillus* species

Microbial strain	Media	Optimum pH	Optimum temp (°C)	PYT activity (IU/mL)	Productivity (IU/mL/day)	Reference
*A. niger* NCIM 563	GrCf^a^ (optimized)	2.5	50	160.0	29.00	Present work
*A. fumigatus* NF 191	PSM (optimized)	(–)	(–)	101.79	25.40	Gangoliya et al. (2015)
*A. oryzae* SBS 50	Starch	5.0	35	15.70	3.90	Sapna (2013)
*A. niger* CFR 335	Potato dextrose broth	(–)	(–)	9.60	1.92	Shivanna and Venkateswaran (2014)
*A. ficuum* SGA 01	Potato dextrose broth	(–)	(–)	8.20	1.64	Howson and Davis (1983)
*A. ficuum* NRRL 3135	MRS medium	(–)	(–)	2.27	0.45	Howson and Davis (1983)
*A. heteromorphus* MTCC 10685	Phytase screening medium (optimized)	6.0	30	24.88	5.00	Lata et al. (2013)

(–) No data reported

^a^GrCf-green chickpea flour

Statistical experimentation for media optimization provides a time saving approach for enhancing PYT production (Bhavsar et al. 2013) and provides the base experimental conditions maintaining/improving the productivity for scale-up. Our results with this approach showed that the PYT productivity obtained in a 10-L fermenter working volume (41.0 IU/mL/day) was improved by a factor of 2.87 and 1.41 times from shake flask (14.3 IU/mL/day) and 2-L scale (29 IU/mL/day) experiments, respectively. The increase in productivity with reduction in production time at 10-L fermenter scale may be due to the better maintenance of fermentation parameters viz., agitation, temperature, aeration, etc. This observation further supports the biotech potential of the present PYT production process. Studies of submerged PYT production using *S. thermophile* in cane molasses medium showed that the productivity obtained in the 10-L working volume fermenter (5.2 IU/mL/day) was improved by a factor of 2.2 times from shake flask (2.5 IU/mL/day) experiments and having optimum activity at pH 5.5 and 45 °C (Singh and Satyanarayana 2008). Similar trend of increased productivity by 1.5 times for glucoamylase production was observed by Kumar et al. (2007).

In conclusion, the present study shows an efficient process of producing PYT, which has wide applications prospects in animal feed and agriculture. Our studies obtained high yields of PYT from *A. niger* NCIM 563 using GrCf. The PYT from *A. niger* was found to be stable over wide range of temperature and pH and has thus shown the necessary potential for use as animal feed supplement as well as on crops for field applications. The PYT from *A. niger* NCIM 563 beneficially shows biodegradation of CPyF when tested on green chillies. Studies show biodegradation of CPyF is maximum at conditions chosen close to the enzyme optimum conditions for high PYT activity (50 °C and pH 2.5). These findings thus bring out an interesting and new applications for PYT produced from GrCf and *A. niger* NCIM 563. Although, the above optimal conditions do not correspond to field, they do justify the need to carry out studies that would improve the efficacy of PYT for biodegradation of OpP compounds.

## Additional files

**Additional file 1.** Additional figures and tables.

**Additional file 2.** Additional data.

## Abbreviations

BBD: Box-Behnken design
CPyF: chlorpyrifos
GrCf: green chickpea flour
MCP: monocrotophos
MP: methyl parathion
OpP: organophosphorus pesticide
PYT: phytase
PBD: Plackett–Burman design
