# Supplementary material for: Phytase production by Aspergillus niger NCIM 563 for a novel application to degrade organophosphorus pesticides
Source: AMB Express. 2017 Mar 21;7:66. doi: 10.1186/s13568-017-0370-9 (PMC5359262; doi:10.1186/s13568-017-0370-9)
Supplement: Supplementary file 1 — Additional file 1. Additional figures and tables. [file 13568_2017_370_MOESM1_ESM.pdf]

Journal – AMB Express (Manuscript Ref. No. AMBE-D-16-00209)

Phytase production by *Aspergillus niger* NCIM 563 for a novel application to degrade organophosphorus pesticides

Parin C. Shah[1, 2] • V. Ravi Kumar[1, 3] • Syed G. Dastager[1, 2] • Jayant M. Khire[1, 2*]

[1] Academy of Scientific and Innovative Research (AcSIR), CSIR - National Chemical Laboratory (CSIR-NCL), Pune, 411008, India.

[2] National Collection of Industrial Micro-organisms (NCIM) Resource Center, CSIR - NCL, Pune, 411008, India.

[3] Chemical Engineering and Process Development Division, CSIR-NCL, Pune, 411008, India.

* Corresponding author: Dr. J. M. Khire
NCIM Resource Centre,
Biochemical Sciences Division,
CSIR– National Chemical Laboratory,
Pune, 411008.
India.
Ph: +91 – 20 – 25902505
Fax: +91 – 20 – 25882647
Email Id: khirejayant@gmail.com

Fig. S1 Studies on optimum temperature and stability of PYT at different temperature

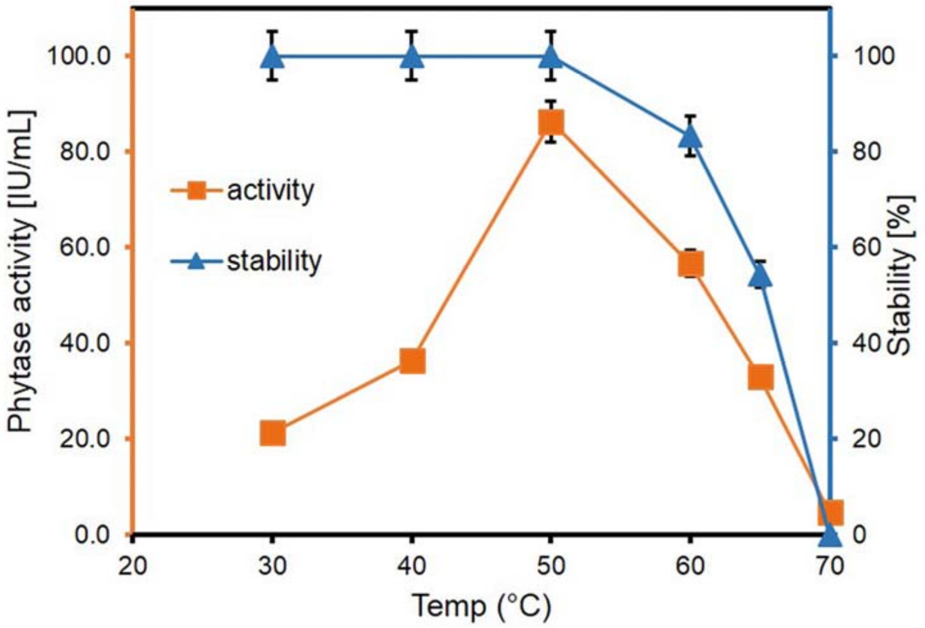

Fig. S2 Studies on optimum pH and stability of PYT at different pH

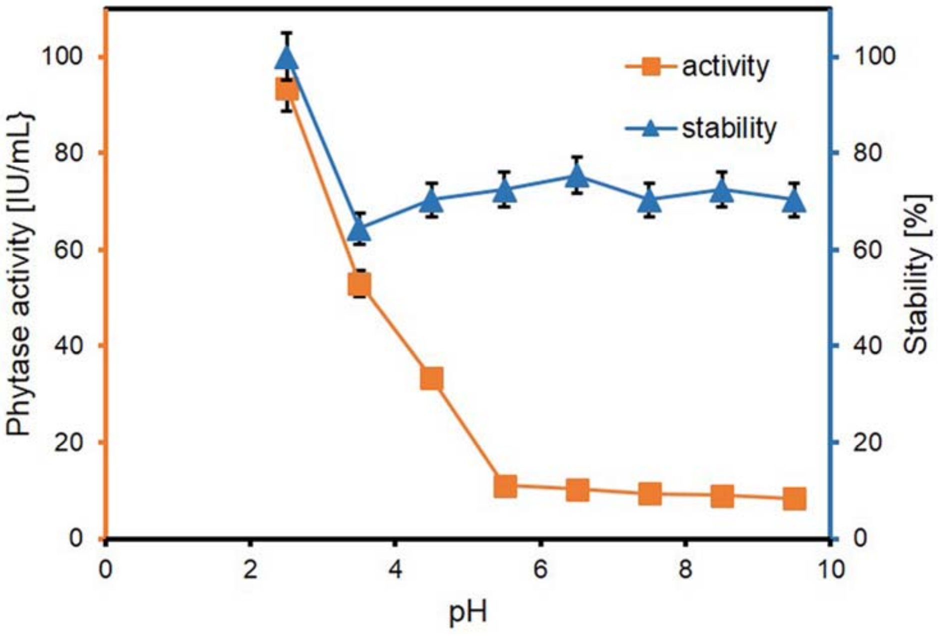

Fig. S3 Efficacy of PYT in stimulated gastric fluid at different pH

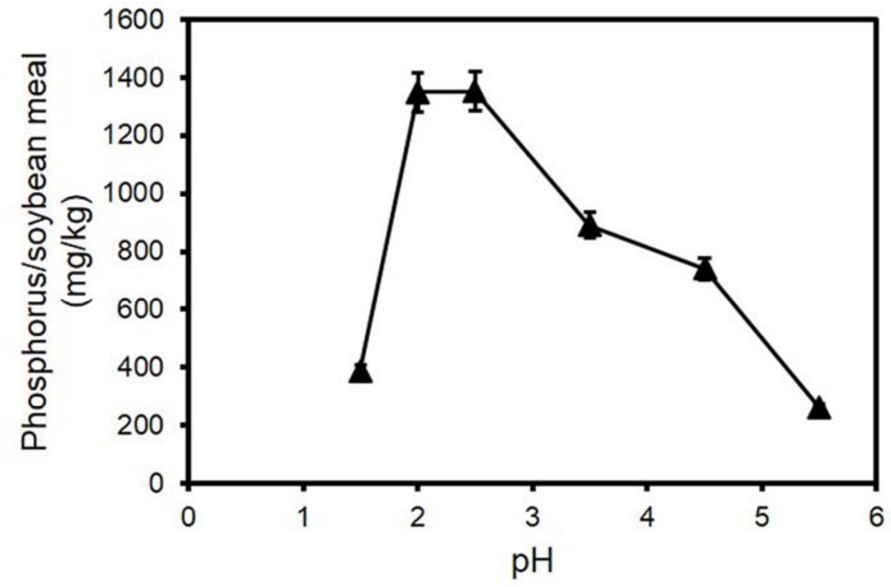

Fig. S4 Degradation of OpP: (a, b) MCP and (c, d) MP using PYT

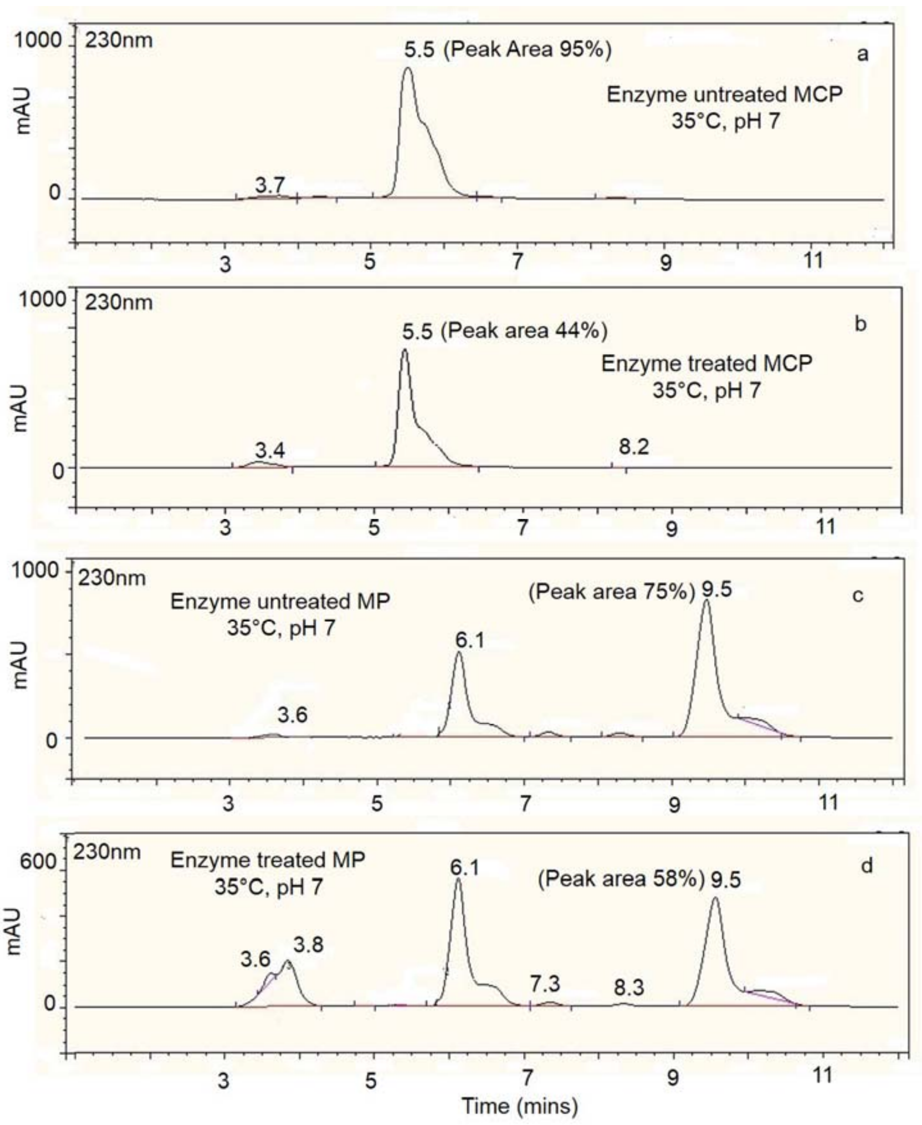

Fig. S5 Degradation of CPyF on harvested green chilli (a) control; (b) using 250 IU PYT

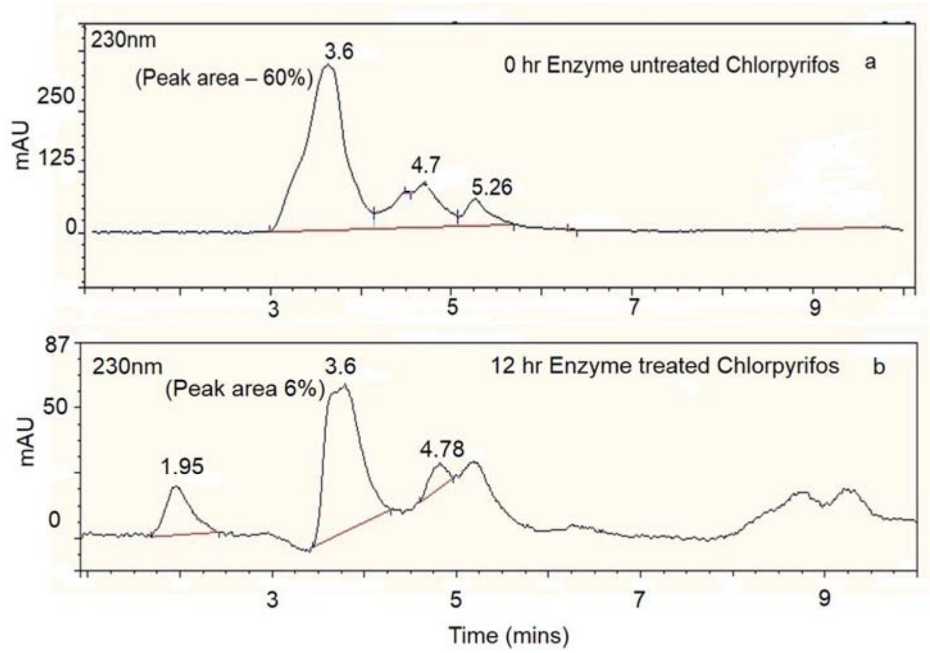

Fig. S6 Pareto chart showing positive and negative effect of significant factors by PBD analysis

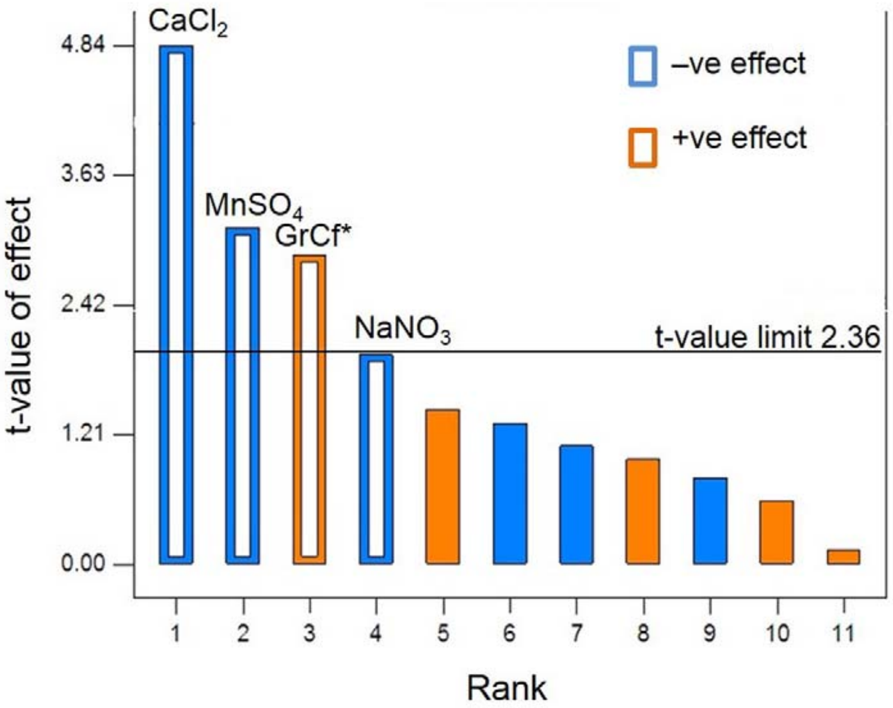

*GrCf- green chickpea flour

Table S1 Selected variables for PBD and their assigned levels for studying PYT production in 100 mL media

S. No.	Code	Variables	Low level (-)	High level (+)
1	A	Glucose (g)	3.00	5.00
2	B	$NaNO_3$ (g)	0.30	0.80
3	C	$MgSO_4 .7H_2O$(g)	0.04	0.10
4	D	KCl (g)	0.04	0.10
5	E	$FeSO_4.7H_2O$ (g)	0.01	0.02
6	F	Tween 80 (µl)	10.00	20.00
7	G	GrCf* (g)	0.75	1.25
8	H	Dextrin (g)	0.20	0.50
9	J	$MnSO_4·H_2O$ (g)	0.005	0.020
10	K	$CaCl_2·2H_2O$ (g)	0.10	0.30

*GrCf – green chickpea flour

Table S2 Selected variables for BBD and their assigned levels for studying PYT production in 100 mL media

Variables	Variable code	Units	Levels		
			-1	0	1
$NaNO_3$	A	g	0.200	0.400	0.600
$MnSO_4{\cdot}H_2O$	B	g	0.005	0.013	0.020
$CaCl_2{\cdot}2H_2O$	C	g	0.050	1.000	0.150
GrCf*	D	g	1.000	1.500	2.000

*GrCf – green chickpea flour

Table S3 PBD for the selected variables along with the experimentally obtained mean PYT production response values**

Run no.	A Glucose g%	B $NaNO_3$ g%	C $MgSO_4 \cdot 7H_2O$ g%	D KCl g%	E $FeSO_4 \cdot 7H_2O$ g%	F Tween 80 %	G GrCf* g%	H Dextrin g%	J $MnSO_4 \cdot H_2O$ g%	K $CaCl_2 \cdot 2H_2O$ g%	PYT activity (IU/mL)
1	5.0	0.3	0.1	0.1	0.02	0.01	0.75	0.2	0.02	0.1	93±4.6
2	3.0	0.3	0.04	0.04	0.01	0.01	0.75	0.2	0.005	0.1	109±5.4
3	3.0	0.8	0.04	0.1	0.02	0.01	1.25	0.5	0.02	0.1	84±4.2
4	3.0	0.3	0.04	0.1	0.01	0.02	1.25	0.2	0.02	0.3	89±4.5
5	3.0	0.3	0.1	0.04	0.02	0.02	0.75	.5	0.02	0.3	33±1.7
6	5.0	0.8	0.04	0.04	0.01	0.02	0.75	0.5	0.02	0.1	61±3
7	3.0	0.8	0.1	0.04	0.02	0.02	1.25	0.2	0.005	0.1	121±6
8	5.0	0.8	0.1	0.04	0.01	0.01	1.25	0.2	0.02	0.3	52±2.6
9	5.0	0.3	0.04	0.04	0.02	0.01	1.25	0.5	0.005	0.3	76±3.8
**10**	**5.0**	**0.3**	**0.1**	**0.1**	**0.01**	**0.02**	**1.25**	**0.5**	**0.005**	**0.1**	**132**±6.6
11	5.0	0.8	0.04	0.1	0.02	0.02	0.75	0.2	0.005	0.3	53±2.7
12	3.0	0.8	0.1	0.1	0.01	0.01	0.75	0.5	0.005	0.3	69±3.5

*GrCf – green chickpea flour

** Experiments were carried out in triplicate along with the type of error analyses (i.e., mean ± SD).

Table S4 BBD for the selected variables along with the experimentally obtained mean PYT production response values**

Run no.	A $NaNO_3$ g%	B $MnSO_4 \cdot H_2O$ g%	C $CaCl_2 \cdot 2H_2O$ g%	D GrCf* g%	PYT activity (IU/mL)
1	0.4	0.005	0.05	1.5	82±4.1
2	0.6	0.013	0.05	1.5	67±3.3
3	0.4	0.013	0.05	1.0	116±5.8
4	0.4	0.013	0.15	1.0	114±5.7
5	0.4	0.020	0.05	1.5	90±4.5
6	0.6	0.013	0.15	1.5	70±3.5
7	0.4	0.020	0.10	2.0	5±0.3
8	0.4	0.020	0.15	1.5	85±4.3
9	0.4	0.013	0.10	1.5	53±2.7
10	0.6	0.013	0.10	1.0	123±6.2
11	0.4	0.013	0.05	2.0	6±0.3
12	0.6	0.013	0.10	2.0	7±0.4
13	0.4	0.013	0.10	1.5	70±3.5
14	0.2	0.013	0.15	1.5	92±4.6
15	0.6	0.020	0.10	1.5	125±6.3
16	0.4	0.013	0.10	1.5	117±5.9
17	0.6	0.005	0.10	1.5	92±4.6
18	0.4	0.005	0.10	2.0	7±0.4
19	0.2	0.013	0.05	1.5	95±4.8
20	0.4	0.013	0.10	1.5	112±5.6
21	0.4	0.005	0.10	1.0	142±7.1
22	0.2	0.013	0.10	1.0	127±6.4
23	0.4	0.013	0.10	1.5	155±7.8
24	0.2	0.020	0.10	1.5	140±7
**25**	**0.4**	**0.020**	**0.10**	**1.0**	**160±8**
26	0.2	0.005	0.10	1.5	134±6.7
27	0.4	0.005	0.15	1.5	56±2.8
28	04	0.013	0.15	2.0	22±1.1
29	0.2	0.013	0.10	2.0	14±0.7

*GrCf – green chickpea flour
** Experiments were carried out in triplicate along with the type of error analyses (i.e., mean ± SD).
